# Supplementary material for: Retrosynthetic reaction pathway prediction through neural machine translation of atomic environments
Source: Nat Commun. 2022 Mar 4;13:1186. doi: 10.1038/s41467-022-28857-w (PMC8897428; doi:10.1038/s41467-022-28857-w)
Supplement: Supplementary file 1 — Supplementary Information [file 41467_2022_28857_MOESM1_ESM.pdf]

Supplementary Information

RetroTRAE: retrosynthetic translation of atomic environments with Transformer

U. V. Ucak et al.

1 Supplementary Figures

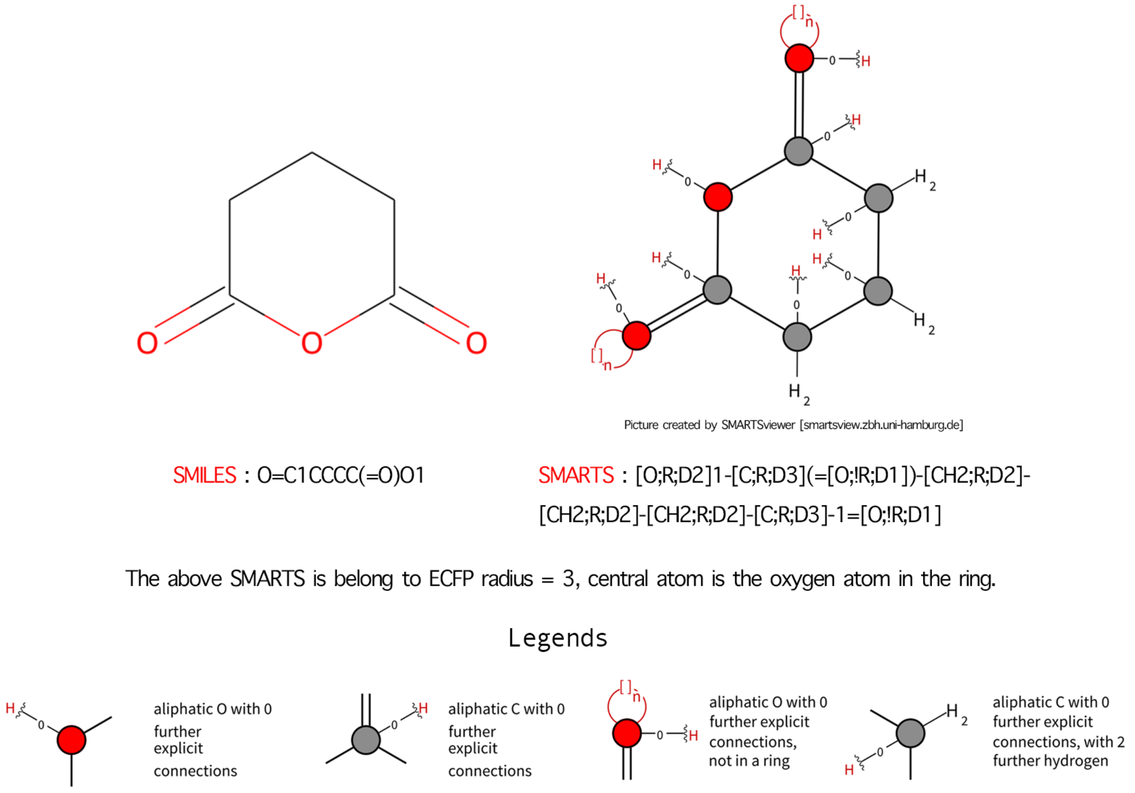

Supplementary Figure 1: Isomorphism between ECFP fragments and a real molecule. A fragment isomorphic to the molecular structure can always be found with a proper choice of fingerprint radius.

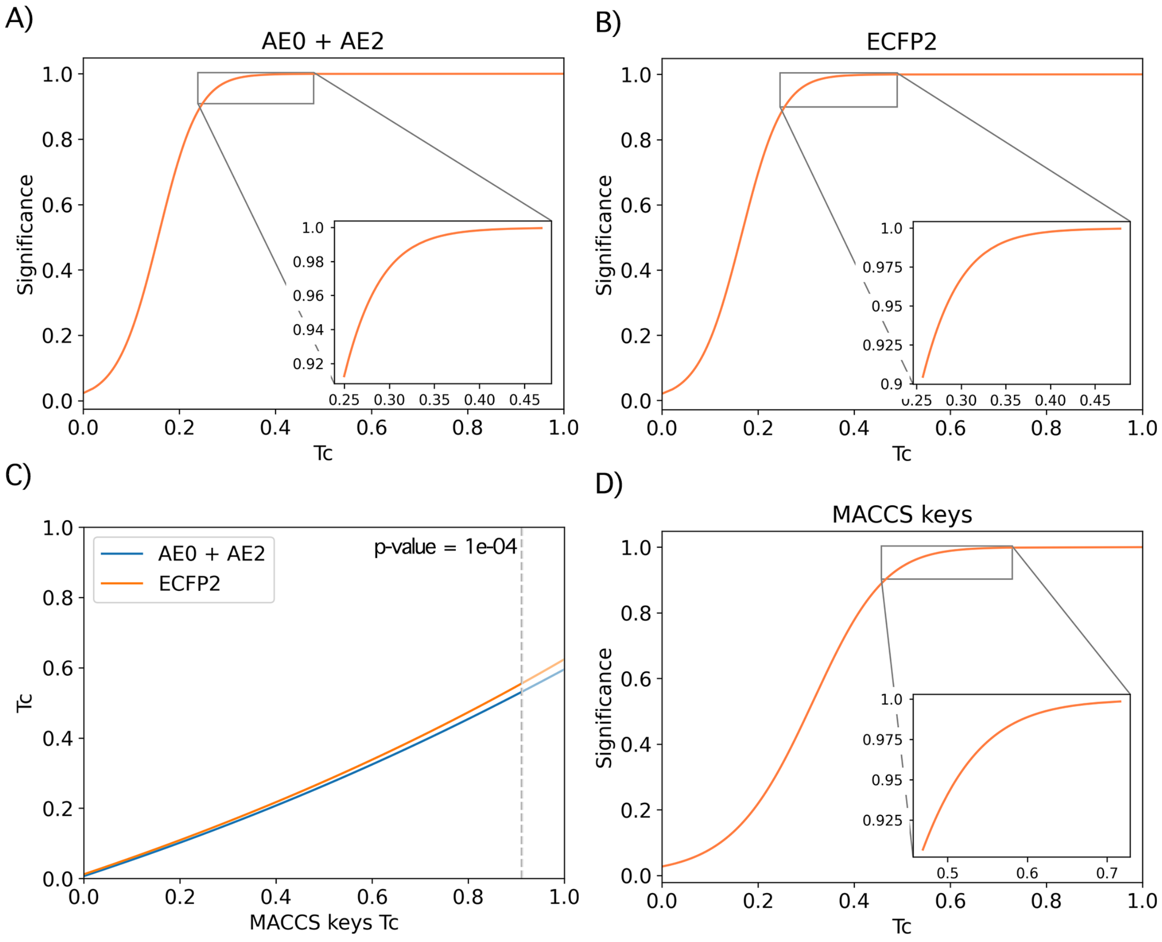

Supplementary Figure 2: Cumulative distribution functions of selected representations. Figures A, B and D represent the cumulative distribution function of the reactants in the USPTO DB for the unified atom environments, ECFP2, and MACCS keys respectively. The measure 1 − (p-value) is used to assess significance. P-values has the range 0 to 1 and smaller p-values indicate higher significance. The Figure D shows the relation of MACCS Tc values to Tc values of unified atom environments and ECFP2. The vertical dashed line corresponds to a significance level of p-value set to 1e-04.

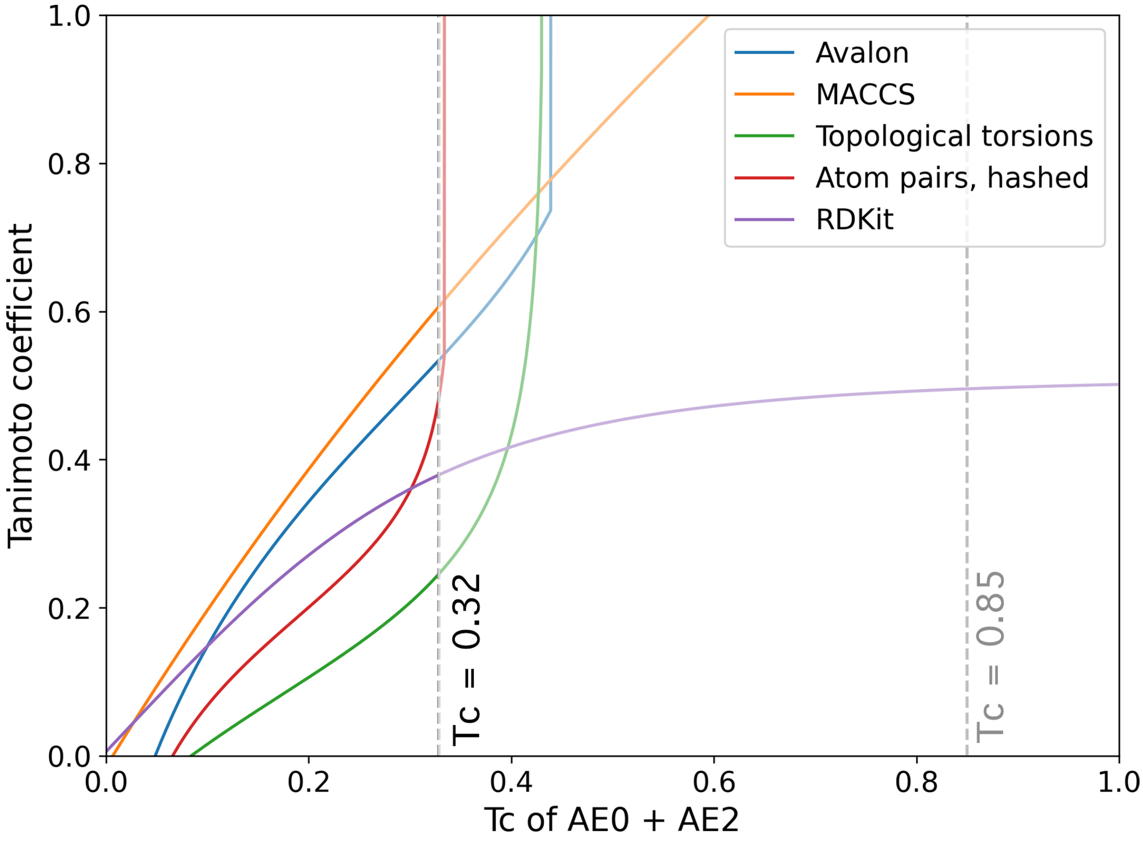

Supplementary Figure 3: The statistical equivalences between the similarity scores of various structural fingerprints. The region beyond the p-value 0.01 is grayed out since the curves are expected to be less reliable. The similarity cut-off of $Tc = 0.85$ corresponds to a very low p-value of 6×10^{-6}.

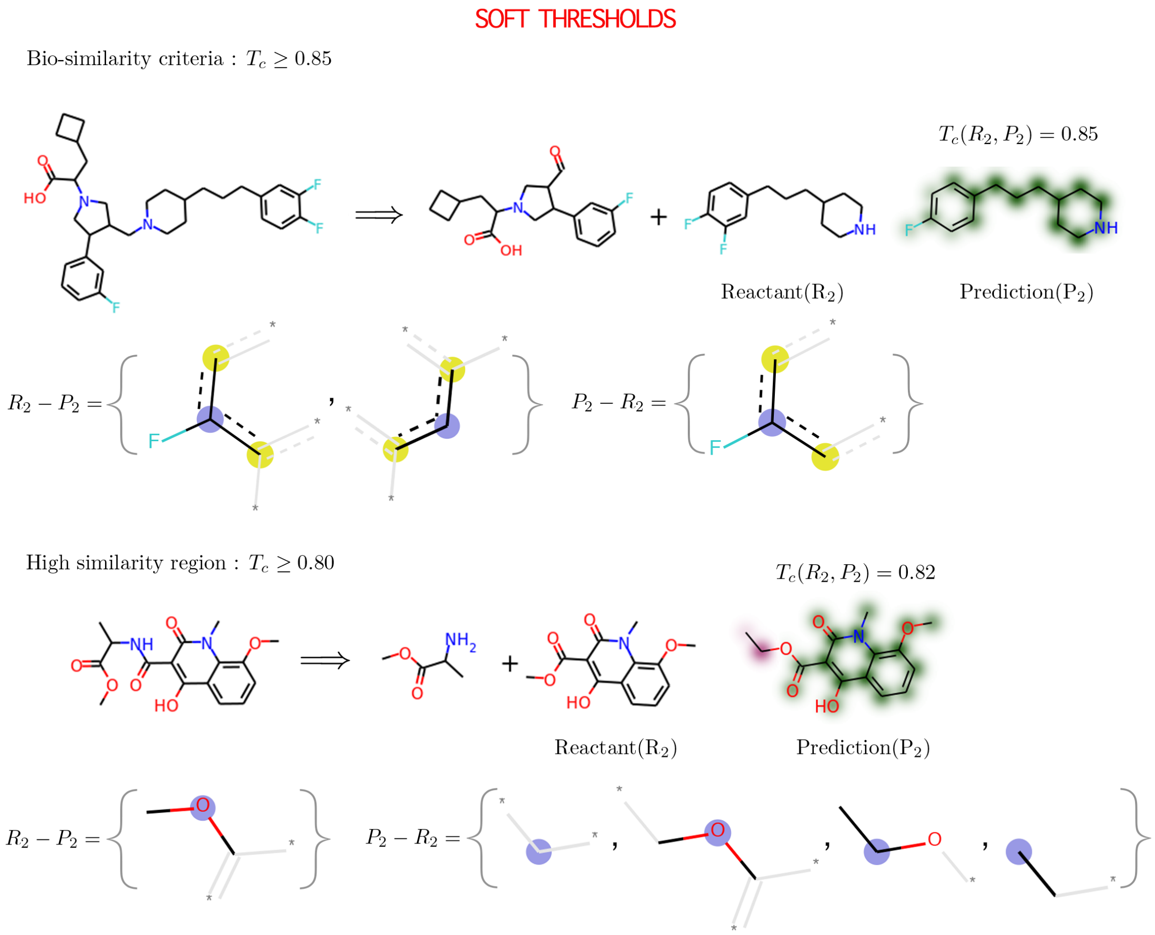

Supplementary Figure 4: A representative example belonging to bio-active, and highly similar predictions is shown. Distinct fragments are given as SMARTS patterns. Predictions are drawn as similarity maps using the Morgan fingerprints. The first reactant is predicted correctly and the qualities of the second reactants are evaluated. The fragments only belonging to the prediction or its true counterpart are given as set notation differences, which allows us to describe the chemical change more concretely. Colors indicate atom-level contributions to the overall similarity (green: increases in similarity score, red: decreases in similarity score, uncolored: has no effect).

2 Supplementary Tables

Supplementary Table 1: Hyper-parameter space and hyper-parameters for the best model.

**Parameter**	**Possible Values**	**Best Model Parameters**
Number of layers	2-8	6
Number of head	4-12	8
Size of hidden layers	256, 512, 1024	512
Size of intermediates	512, 1024, 2048	2048
Optimizer	Adam or SGD	Adam
Dropout	0.1, 0.2, 0.4	0.1
Learning rate	0.0001 - 0.002	0.0001 - 0.002
Learning rate scheduler	Cyclic LR, SGDR	Cyclic LR

Supplementary Table 2: Token statistics: Sequence length and vocabulary size statistics

Representation	Sequence length		Vocabulary Size	
	Source	Target	Source	Target
MACCS	32.30	39.15	130	131
ECFP0	9.95	13.44	79	99
AE0	9.95	13.44	119	118
ECFP2	18.33	21.37	1025	1028
AE2	18.33	21.37	7533	8007
ECFP4	46.39	52.78	2052	2053

Supplementary Table 3: Similarity values of hard thresholds based on sequence length. The single and double mutant cases as a function of reactant fingerprint length

Length	5	8	11	14	17	20	23	26	29	32
T_c of SM	0.80	0.88	0.91	0.93	0.94	0.95	0.96	0.96	0.97	0.97
T_c of DM	0.60	0.75	0.82	0.86	0.88	0.90	0.91	0.92	0.93	0.94

Supplementary Table 4: Probability of finding extremely close neighbours. The results are based on the CDF generated by using 1.3 million compounds using AE0 + AE2.

Thresholds	10%	1%	0.1%	0.01%	0.001%
Tanimoto metric	>.24	>.33	>.42	>.53	>.76

Supplementary Table 5: AE vs substructure based fingerprints : Quantitative similarity score comparison of MACCS, RDKit and AE representations within the high similarity regime tested on single and double mutated predictions of RetroTRAE.

Fingerprint type	T_c of SM	T_c of DM	$T_c = 1.0$	Average
MACCS	0.99	0.99	17	0.99
RDKit	0.99	0.95	3	0.97
AEs	0.94	0.88	0	0.91

Supplementary Table 6: Results of data augmentation (x10) and with/without positional encoding trained with Karpov's cyclic learning scheduler strategy.

	Unimolecular			Bimolecular		
	$T_c = 1.0$	$T_c >= 0.85$	$\overline{T_c}$	$T_c = 1.0$	$T_c >= 0.85$	$\overline{T_c}$
Positional encoding	55.4	68.1	88	58.3	63.4	77
No Positional encoding	53.9	66.5	87	56.1	61.7	76
x10 Aug (products only)	56.4	68.2	88	60.1	64.3	79
x10 Aug (products+reactants)	44.1	64.0	84	-	-	-

3 Supplementary Notes

Supplementary Note 1 : Raw data for Figure 5 in main manuscript titled as the Area-proportional Euler graph representing the space of atomic environments is given below. The data contain the number of unique AE0 and AE2 in each database and their intersections.

1. USPTO-AE0 = 275,
2. ChEMBL-AE0 = 386,
3. PubChem-AE0 = 3450,
4. USPTO-AE0 $\cap$ ChEMBL-AE0 = 171,
5. USPTO-AE0 $\cap$ PubChem-AE0 = 250,
6. ChEMBL-AE0 $\cap$ PubChem-AE0 = 358,
7. USPTO-AE0 $\cap$ ChEMBL-AE0 $\cap$ PubChem-AE0 = 170,
8. USPTO-AE2 = 15982,
9. ChEMBL-AE2 = 39149,
10. PubChem-AE2 = 533276,
11. USPTO-AE2 $\cap$ ChEMBL-AE2 = 10251,
12. USPTO-AE2 $\cap$ PubChem-AE2 = 15224,
13. ChEMBL-AE2 $\cap$ PubChem-AE2 = 37725,
14. USPTO-AE2 $\cap$ ChEMBL-AE2 $\cap$ PubChem-AE2 = 10232,
